# The brain timewise: how timing shapes and supports brain function

**DOI:** 10.1098/rstb.2014.0170

**Published:** 2015-05-19

**Authors:** Riitta Hari, Lauri Parkkonen

**Affiliations:** Department of Neuroscience and Biomedical Engineering, Aalto University, FI-AALTO 00076, Espoo, Finland

**Keywords:** brain imaging, hierarchical order, magnetoencephalography, networks, prediction, time

## Abstract

We discuss the importance of timing in brain function: how temporal dynamics of the world has left its traces in the brain during evolution and how we can monitor the dynamics of the human brain with non-invasive measurements. Accurate timing is important for the interplay of neurons, neuronal circuitries, brain areas and human individuals. In the human brain, multiple temporal integration windows are hierarchically organized, with temporal scales ranging from microseconds to tens and hundreds of milliseconds for perceptual, motor and cognitive functions, and up to minutes, hours and even months for hormonal and mood changes. Accurate timing is impaired in several brain diseases. From the current repertoire of non-invasive brain imaging methods, only magnetoencephalography (MEG) and scalp electroencephalography (EEG) provide millisecond time-resolution; our focus in this paper is on MEG. Since the introduction of high-density whole-scalp MEG/EEG coverage in the 1990s, the instrumentation has not changed drastically; yet, novel data analyses are advancing the field rapidly by shifting the focus from the mere pinpointing of activity hotspots to seeking stimulus- or task-specific information and to characterizing functional networks. During the next decades, we can expect increased spatial resolution and accuracy of the time-resolved brain imaging and better understanding of brain function, especially its temporal constraints, with the development of novel instrumentation and finer-grained, physiologically inspired generative models of local and network activity. Merging both spatial and temporal information with increasing accuracy and carrying out recordings in naturalistic conditions, including social interaction, will bring much new information about human brain function.

## 1. Introduction

Accurate timing is essential for human brain function. Still, current human brain imaging is dominated by methods focusing on the spatial distributions of brain activity by means of functional magnetic resonance imaging (fMRI) and positron emission tomography (PET). Although these methods are intrinsically sluggish, they have been extremely informative in unravelling brain areas involved in different types of processing. Recent development is moving the focus from single brain areas to dynamic networks whose nodes and connection strengths can change over time.

Less attention has been paid to temporally accurate recording methods, such as magnetoencephalography (MEG) and electroencephalography (EEG), which we discuss in this paper. We first describe temporal integration windows that govern human brain function and their changes in brain disorders. We continue by pondering what kind of traces the temporal dynamics of the environment has left during evolution of the mammal brain. We then discuss the kind of timing information that we can obtain with the current brain imaging tools, and we end by speculating future developments in this field, especially related to our own work on time-resolved MEG imaging of two-person recordings in the study of the brain basis of social interaction and of other human cortical functions.

## 2. Multiple time scales in brain function

### (a) Perception, action and cognition

The relevant time scales of human perception, action and cognition vary considerably [1]. The analysis of directional hearing cues requires resolving temporal differences of just 50 ms, corresponding to 20 kHz, which is a factor of 1000 higher than the frequency of the typical brain rhythms occurring at 10–20 Hz (cycle durations 50–100 ms). The strength of these rhythms is modulated by external stimuli or tasks at rates slower than approximately 2 Hz (approx. 500 ms). The now very popular resting-state fluctuations of brain activity are slower than approximately 0.2 Hz (approx. 5 s). Acute emotions, such as anger and happiness, can arise within seconds, but for mood changes the proper time scales are in the range of minutes. Thus, the time scales of interest vary from microseconds to subseconds, seconds, minutes and even years, resulting in a multitude of ultradian, circadian and annual rhythms; besides behaviour, many of these rhythms can be seen at the level of brain signals [1,2].

Millisecond timing is needed for movements and perception, for example during dancing to the rhythm of music. However, sensory processing in some conditions tolerates inaccuracies up to hundreds of milliseconds. For example, multisensory asynchrony is permitted up to 100–250 ms (approx. 4–10 Hz), meaning that stimuli arriving at intervals less than about 250 ms are perceptually fused to the same object or event. Different tasks have different temporal windows but also different requirements of accuracy and error tolerance.

The brains are able to make temporally accurate predictions of the consequences of the subject's own actions; millisecond-range temporal precision is realized via the efference copies (via corollary discharges) that inform the sensory cortices about the motor plans. For example, activity in human somatosensory cortex differs when the subject touches herself compared with an identical stimulus delivered by someone else, and this difference is observable already about 30 ms after the touch [3]. Similarly, learned regularities in sensory input can give rise to temporal predictions with subsecond accuracy, as is reflected in, for example evoked responses in the auditory cortex to omitted sounds, occurring within 200 ms from the time of the expected sound occurrence [4], or as changes of brain rhythms to support prediction of timing of external events [5,6].

### (b) Hierarchy of time scales

Naturalistic visual [7] and auditory [8] stimulation have been applied during fMRI scanning to reveal a hierarchy of time scales from a few seconds to tens of seconds, expressed as topographically organized maps where the time windows are longer the longer is the area's distance from the early projection cortex. MEG studies, based on the recovery rates of evoked responses—with their sequences of different deflections each reacting differentially to changes in stimulus repetition rate—have pointed to a hierarchical order of much shorter time intervals and demonstrated that one brain area can be, at the same time, involved in processes spanning different time scales [1].

The brain apparently operates in parallel on several timescales which are often nested and hierarchically organized. The functional hierarchy may emerge as the result of self-organization in a system containing multiple time scales, as can be deduced from a network model controlling the physical body and the movements of a humanoid robot [9]. Quite striking evidence of nested time windows exists for cortical mechanisms underlying speech perception, with different integration windows for consonants (20–50 ms), syllables (200–300 ms) and sentences [10,11].

Several bodily rhythms are coupled to each other. For example, the breathing rhythm has been suggested to serve, during evolution, as a common clock for integration of multiple orofacial senses, such as sniffing-related smell and vibrissae-movement-related touch [12]. Respiration as a fundamental function could also be one of the evolutionary bases for the rhythmicity of speech. The rhythmical structure is highly similar in human speech and monkey auditory calls, and in both species the acoustic envelopes and the mouth movements resemble each other at frequencies lower than about 10 Hz [13], that is in the frequency range where abolition of the temporal fluctuations of sounds and the temporal modulations of their frequency patterns most seriously disrupts speech understanding [14].

Generally, cross-frequency coupling of brain oscillations is considered an important coordinating and integrating mechanism of brain activity (e.g. [15,16]). It is a mechanism for the global slow oscillations (usually their phase) to modulate the local fast oscillations (usually their amplitude). The commonly used analysis tools have, however, been recently criticized, because of the easy appearance of spurious cross-frequency coupling [17].

The neurophysiological mechanism supporting such coupling could be that the synaptic oscillatory signal modulates the length constant of neurons of the target area, which in turn affects local spatio-temporal integration of that area's other synaptic inputs in a nonlinear manner—in other words changing the electrotonic size of the pyramidal neuron—and thereby makes the neuron's output vary in phase of the driving rhythm [18].

### (c) Timing in brain disorders

Characteristic of many brain disorders is slowing of the background EEG/MEG rhythms as well as delays in sensory processing as indicated by prolonged latencies of evoked responses. For over 80 years, abnormalities of EEG rhythms and the appearance of abnormal EEG signals have been used as diagnostic tools of various brain disorders [19], although the findings are often quite unspecific.

Clumsy and slow movements are often related, besides lesions of the corticospinal pathways, to disorders of the cerebellum and basal ganglia; for example, bradykinesia (slowed-down movements) in Parkinson's disease is associated with changes in dopamine-dependent rhythms covering a wide frequency range [20].

Timing deficits have been much discussed and debated in dyslexia because the subjects are often slow in reacting to stimuli presented in rapid succession. One frequently presented hypothesis is weakness of the magnocellular pathways that normally transmits impulses to the cortex about 10 ms earlier than the other fibres [21], or the sensory slowing could be secondary to sluggish attention shifting [22]. Whatever their origin may be, these timing disorders contribute to but apparently do not exhaustively explain dyslexia.

## 3. Temporal constraints for brain architecture

### (a) How temporal dynamics was impregnated into our brains

An interesting question is how the different temporal scales have emerged in the human brain during evolution and ontogeny. Evolutionary pressure has arisen from the necessity of the organism, for its survival and reproduction, to perceive and act in the dynamical environment. Additional temporal constraints have arisen from the need to communicate.

Nature is full of concurrently active time scales to which organisms have to adapt and be able to react. Importantly, these basic environmental constraints are quite similar in all mammals living on land. The most evident consequences are the annual and circadian rhythms in various bodily functions, but in a similar way the dynamical environment with all its time scales is the apparent driving force for the hierarchical organization of both temporal and spatial scales in brain activity [1].

When facing the same force, larger masses move or oscillate slower than smaller ones, as dictated by Newton's second law. Think, for example, how differently the wind moves the trunk and the small branches of a tree. Thus, a general inverse relationship exists between the speed of passive responses (and active movements) and the size of real-world objects (and organisms). Physical quantities reflecting such random motion typically manifest a 1/*f* spectrum (corresponding to ‘pink noise’) where the power and frequency of the signal are inversely related. This kind of a relationship, generally called a powerlaw, is omnipresent in the physical world and also common in biological systems. It is thus to be expected that the long evolutionary exposure to the dynamical environment would have led the small spatial scales and short temporal windows to coexist in the same brain regions, similarly to how they coexist in the physical environment. The above analyses of temporal and spatio-temporal windows [1,7,8] support this prediction.

### (b) Preservation of brain rhythms across species

The importance of timing for brain function becomes evident from the surprising preservation of the frequencies of brain rhythms across mammals, in whom the brain volume varies by a factor of 17 000 as recently reviewed [23]. Such regularities are evident across species although, at least in humans, the detailed frequencies and the reactivity of brain rhythms have a clear genetic component as well [24]. This similarity of brain rhythm frequencies is in strong contrast to for example breathing rhythm and heart rate, which scale inversely with animal size.

According to Buzsaki *et al*. [23], the reasons for the brain-rhythm similarity could be related to intrinsic biophysical properties of neurons and microcircuits involved in the generation of the rhythms. These processes impose the temporal limits for synchrony that as such are closely related to the timing between pre- and postsynaptic firing (and thereby synaptic plasticity) as well as to second-messenger processes that make the plastic changes permanent.

Another interesting constraint, also mentioned by Buzsaki *et al*. [23], is the mechanics of the effector systems, especially the contraction mechanisms of skeletal muscles where the properties of myosin and actin filaments, and especially their interaction (contraction) speed, have remained largely similar across mammals. Consequently, both the input and output systems of all animals equipped with these physiological ‘devices’ need to keep approximately similar timing.

In other words, the temporo-spatial properties and regularities of the environment have, during evolution, shaped both the sensory and motor systems, and less directly also the executive functions that are temporally constrained by sensing and acting.

### (c) How to wire a brain

It is beneficial for an animal to react to danger or a threat as rapidly as possible, thus relying on fast transmission channels. The conduction velocity of an axonal fibre depends on the fibre diameter: in a myelinated axon the velocity increases linearly as a function of the diameter, whereas in an unmyelinated fibre it increases only as the square root of the diameter. Although thin unmyelinated fibres are metabolically expensive (because of the increased capacitance of the membrane compared with myelinated fibres) they still are very common in the brain. One may thus wonder—given the importance of accurate timing and speed for survival—why the brain spends energy on such thin, slowly conducting fibres.

Estimating from the fibre diameter distribution of the human corpus callosum [25], time lags between the hemispheres vary from 3 to 300 ms. Very strikingly, in all mammals the shortest transmission times between the hemispheres—independently of the brain size—remain about the same, shorter than 5 ms [26], because a fraction of the thickest fibres scales with brain size.

It is evident that one cannot keep accurate timing in large mammal brains just by increasing the diameter of all axons, as such a design would lead to a catastrophic increase of the brain size [27], making delivery impossible. The required high-speed information transfer is thus realized by a significant increase in the diameter of only a small fraction of axons in the corpus callosum, which is enough to keep the delays short without spending too much of the expensive brain volume. Evolution has, however, preserved the slow but dense thin fibres, thereby guaranteeing good connectivity at a compromised speed.

An interesting parallel is seen in the current development of interconnections on semiconductor chips where space is also at a premium [28]: a large number of thin (slow) connections form a dense local wiring on the chip, whereas thick (fast) wires are used for global communication on the chip. Similarly to in the brain, the connections in semiconductor integrated circuits can be either fast or dense but not both at the same time without leading to problems of packaging and space. Thus, similar compromises have taken place both in chip design and in the evolution of the mammal brain.

Many studies and theoretical treatises consider the brain as a small-world network, where long-range connections are sparse and contacts between distant areas come as a surprise. The presence of small-world network structure has been recently challenged at local scales, because retrograde tracing has demonstrated several clusters of connections that have not previously been seen with other methods [29], thereby suggesting that the cortical connectivity matrix can be very dense at local scales [30]. At the level of the whole brain, however, the connectivity is predicted by an exponential distance model where connections are lognormally distributed, with short connections weighted strongly but long connections available to connect distant areas [31]. This view of rare long-distance connections fits nicely with the above discussion about the fibre-thickness distributions.

We have previously suggested [1] that the dispersion of the interhemispheric timing because of the wide distribution of conduction velocities in the connecting fibres is useful also in keeping the system flexible, as it prevents excessive ‘neurons that fire together, wire together’ phenomenon that would otherwise freeze and dedifferentiate the system. Cortical dedifferentiation has been demonstrated in monkeys exposed to repetitive monotonous hand movements so that the resulting clumsiness resembled occupational repetitive-strain syndrome [32].

## 4. Time-resolved brain imaging

### (a) Basics of magnetoencephalography and electroencephalography

Monitoring neuronal activity at the speed it occurs requires measuring the electric activity within neurons. While several invasive techniques exist to perform these measurements both *in vitro* and *in vivo*, MEG and EEG are currently the only non-invasive ways to record electric activity of neuronal populations. The bulk of MEG and EEG signals are generated by postsynaptic currents in the apical dendrites of cortical pyramidal neurons; in EEG, we record the potential distribution caused by these currents on the scalp, whereas in MEG we collect the information by measuring the tiny magnetic fields produced by the currents (for reviews, see [33,34]).

**Figure 1.**
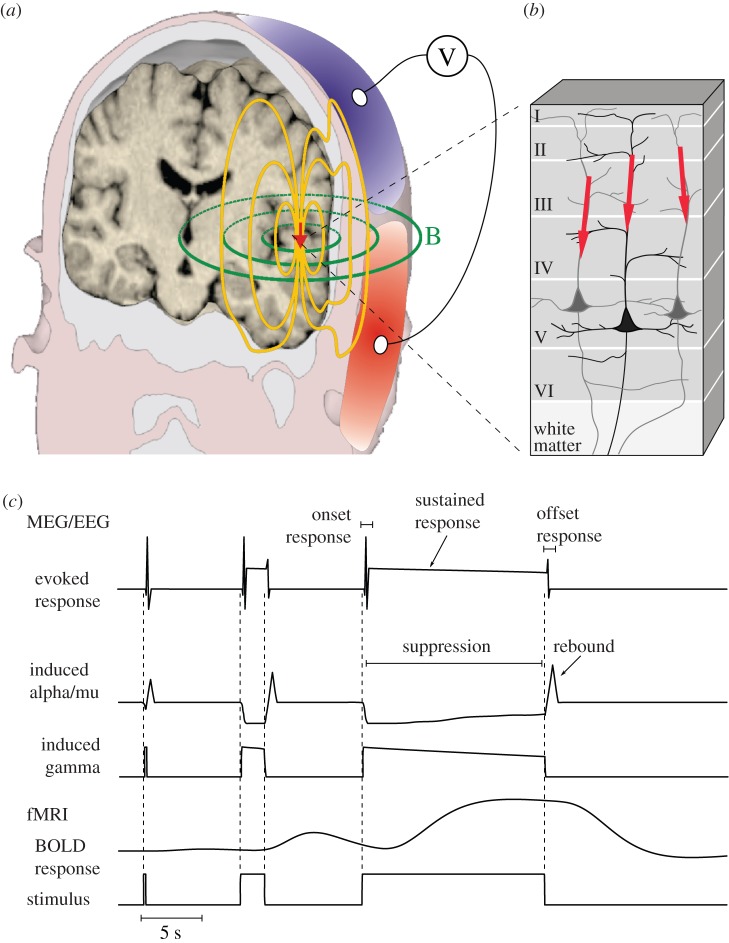
Genesis of EEG and MEG signals. (*a*) Electric currents (red arrow) in active neurons drive volume currents (yellow lines) within the head, which gives rise to a potential distribution (V) on the scalp. The currents also generate a magnetic field (green lines; B) outside of the head; here the direction of the magnetic field follows (according to the right-hand rule) the direction of the net intracellular currents. (*b*) The main contribution to EEG and MEG signals comes from post-synaptic currents (red arrows) in the apical dendrites of pyramidal neurons. (*c*) A highly schematic illustration of electrophysiological (MEG/EEG) and haemodynamic (fMRI) response time courses to stimuli of three different durations. Evoked responses are phase-locked to the stimuli while induced responses reflect amplitude changes in the non-phase-locked oscillatory brain activity.

MEG is currently performed almost exclusively with superconducting SQUID (superconducting quantum interference device) sensors that have the exquisite sensitivity to pick up the weak, extracranial neuromagnetic fields. Yet, for a measurable signal, synchronized activation of tens of thousands of nearby neurons is needed, both because the signals are tiny and because currents into opposite directions within the cortex result in considerable cancellation (figure 1*a,b*). Response averaging is often necessary to uncover the signal of interest amidst noise due to instrumentation, biological artefacts and background brain activity.

MEG and EEG excel in picking up transient evoked responses as well as rhythmic brain activity (figure 1*c*). However, with MEG and EEG, it is typically difficult to record slowly changing brain signals that are associated, for example, with slowly rising sounds or with cognitive tasks that are not driven by temporally accurate external stimuli. This problem can be alleviated to some extent by using continuous dynamic stimuli crafted to elicit evoked responses at a distinct frequency. Such a ‘frequency tagging’ approach allows to probe so slowly changing brain states that they as such could not provide temporally accurate triggers for analysis of brain signals that are time-locked to certain events, for example, the perceptual states associated with ambiguous visual stimuli [35].

Both MEG and particularly EEG sensor signals are a mixture of several neural sources, and to disentangle their timing source modelling is needed. Thus, even if accurate localization of the activity is not the main target, high spatial resolution is beneficial as it allows spatial separation between signals arising from different sources. MEG performs considerably better than EEG in this respect, which has implications for studies of functional connectivity between brain regions. Yet, the limited spatial resolution may give rise to spurious functional connectivity unless the estimation excludes in-phase correlations. Similarly, *a priori* selection of regions-of-interest, as opposed to performing full-brain or whole-cortex analysis, can give misleading results as the activity of the sources not accounted for in the model may leak in to the estimated time series of the selected areas.

MEG, similarly to fMRI, is employed increasingly to explore connectivity, causality and network structure. However, the whole brain imaging community still has problems with proper functional connectivity measures since the widely applied measures tell about correlations, not about causal relationships. Data-driven analyses are beneficial in classifying responses or ‘decoding’ mental contents from the responses. For example, single-trial MEG responses seem to contain information about low-level visual features (here spatial frequencies of gratings) already at the time of the earliest cortical evoked responses, at about 50 ms [36].

### (b) Limitations of the current magnetoencephalography/electroencephalography approaches

Even with electrophysiological methods we are biased to responses attributable to the fastest fibres, whereas the signalling in the slower ones is very difficult to detect because of the larger temporal dispersion. A robust example is the compound action potential of a peripheral nerve that is dominated by the signals from the fastest (thickest) myelinated fibres and only after averaging some contributions can be seen from the slower conducting myelinated fibres, and signals from unmyelinated fibres typically remain invisible. Thus, there is more happening than our recordings can tell.

Even single-unit recordings directly from the monkey cortex may not clarify the activation order of different brain areas: despite a general progression from anatomically lower to higher visual cortices in monkeys, the firing is temporally overlapping [37], and it is thus difficult to specify temporal boundaries for a hierarchy of brain areas. Normal causality measures or even partial coherence measures typically fail if the communication between the brain areas overlaps heavily in time. A further problem is that most brain areas have reciprocal connections, which still hampers the directionality analyses. However, it might be possible to separate incoming and outgoing signalling, at least to some extent, because the feedback and feed-forward connections, landing to different depths in the cortex, employ different frequencies [38]. In macroscopic brain imaging experiments, especially those carried out with fMRI, a common approach to look at the connectivities of different brain regions is dynamical causal modelling which can tell about the directions of information flow, given a predetermined connectivity pattern, but naturally not about the cortical layers that are involved [39].

Despite their good agreement in studying sensory projection cortices (e.g. [40]), MEG/EEG and fMRI often imply different spatial patterns for brain activation even in similar cognitive tasks and in the same subjects (e.g. [41]). It has been suggested that one should first find the activated areas (blobs) with fMRI and then use MEG or EEG to determine the temporal relationships between these areas. However, this approach would work only if the physiological sources of both MEG/EEG and fMRI were identical. Discrepancies between the methods are to be expected to some extent as fMRI reflects neural activity only indirectly, via the BOLD (blood oxygenation-level dependent) signal arising from neurovascular coupling, whereas MEG/EEG pick up signals directly related to the neuronal activity (for time courses, see figure 1*c*). Another apparent difference between the methods, not yet discussed in the literature as far as we know, is that MEG/EEG weights strongly the fastest-conducting pathways, whereas fMRI probably receives its main contribution from neuronal ensembles that are connected via slow and thin fibres, thereby apparently reflecting functionally different brain activations. This difference further emphasizes the complementary nature of these methods.

### (c) Future methods for non-invasive time-resolved brain imaging

How could we improve non-invasive time-resolved imaging of the human brain? First of all, the measurements should be performed as close to the neural generators as possible. However, with an intact skull, the distance from the outside of the head to the closest sources in the cortex is at least 1.5 cm, which sets an upper bound for the spatial frequencies (how fast the signals change in space) and thereby for the resolution any MEG or EEG sensor array can provide. In the present-day SQUID-based magnetometers, the sensors are as far as 4–5 cm from the most superficial sources. Since MEG picks up signals mostly from sources in fissural cortex, the sensor-to-source distance can approach even 7 cm. In children, when measured with the current adult-head-optimized devices, these numbers are even larger if both hemispheres are to be measured simultaneously. However, a few MEG systems designed for infant heads are already available. Moreover, by recording just one hemisphere at a time with an adult MEG device on which the baby's head rests, the distances to sources are efficiently minimized.

The large distances are mostly due to the very low temperatures required by the SQUID sensors; the necessary thermal insulation for a vessel containing liquid helium (4 K = –269°C) typically occupies at least 2 cm and makes it impossible to fabricate arrays adaptable to the head size and shape. Thus, disposing of the cryogenics would allow sensors closer to the cortex. To move in this direction, one could employ SQUIDs made of high-critical-temperature superconducting materials, which enable operation at liquid nitrogen (*T* = 77 K = –196°C) temperatures and possibly allow for an adaptable sensor helmet. However, the sensitivity of the current high-Tc SQUIDs is an order of magnitude worse than that of their liquid-helium-cooled low-Tc cousins (typically 3 fT/√Hz in the white-noise region) used in today's MEG systems. This limitation has prevented the widespread use of high-Tc SQUIDs for MEG even though it has already been shown that MEG signals can be recorded with high-Tc SQUIDS [42].

Optically pumped magnetometers (OPMs), also known as atomic magnetometers, are another viable alternative to low-Tc SQUIDs. In particular, the spin-exchange relaxation-free OPMs provide sensitivities comparable to those of the best low-Tc SQUIDs [43] and they have indeed been demonstrated to be capable of recording MEG [44,45]. Since OPMs do not require cooling, but only moderate heating (and the heat can be easily insulated), they can be placed right on the scalp, much like EEG electrodes on an EEG cap. However, the dynamic range and frequency response of OPMs are not yet optimal for MEG.

Both of these technologies would allow magnetic-field measurements within millimetres from the scalp, which would increase the spatial resolution of MEG provided that the noise level of the sensors remains sufficiently low. Yet, the thickness of the intervening skull imposes a hard limit on spatial resolution, and it is therefore unrealistic to assume that MEG could ever delineate simultaneously active cortical regions within a few millimetres, unless very specific and accurate neurophysiological models are available. To fully exploit the more proximal measurements and thus the potential for capturing higher spatial frequencies, the number of channels should be increased compared with today's MEG systems with about 300 channels. However, the remaining about 15-mm distance to the nearest sources reduces the benefit of having sensors packed more densely than about every 15 mm.

There is still room to improve the noise level of the magnetic sensors, including low-Tc SQUIDs, but would that help? At low frequencies (below, say, 50 Hz), the background brain activity typically exceeds the instrumentation noise by an order of magnitude and therefore improvements in sensitivity do not readily translate into improvements in spatial resolution. However, within the high gamma band (more than 60 Hz) and above, the situation is quite different as no prominent brain rhythms exist at those high frequencies. In spectral density, spontaneous brain activity seems to be on par with instrumentation noise at around 100 Hz and then falls below it at higher frequencies. Therefore, further reduction of sensor noise shall improve the visibility of high-frequency responses, particularly of the high gamma band. The fundamental limit is set by the noise due to the thermal motion of charged particles in the body, which is estimated to be about 0.2 fT/√Hz [46].

Sources in deep brain structures are generally poorly visible in MEG. In special cases that allow excessive trial averaging, responses from for example the auditory brainstem can be recorded and their sources identified [47]. Placing the sensors on the scalp as described above does not substantially improve the detectability of these deep sources, although it remarkably improves the detection of the superficial sources, since the signal attenuation happens largely as cancellation by the fields due to the volume currents (J. Iivanainen, M. Stenroos & L. Parkkonen 2014, unpublished data). On the other hand, lowering the noise level of the sensors would be beneficial provided that cortical activity at similar frequencies does not mask the signals from deep sources.

Action potentials, as opposed to postsynaptic currents, are very poorly visible in MEG and EEG due to their quadrupolar sources and low probability for synchronous appearance during the short duration of the potential. However, action potentials at axon bends and extracellular conductivity boundaries show a dipolar component whose signal could be measured at a distance (and has been done so in peripheral nerves). Yet, the problem of lacking temporal synchronization remains, and concerted action potentials have been non-invasively detected only in a few cases (at frequencies around 600 Hz), but if future developments in instrumentation greatly increase the sensitivity at high frequencies (200–2000 Hz), rather small groups of axons with near-simultaneous action potentials could be detected non-invasively. This possibility would substantially expand our window to investigate timing in the brain, allowing for example the evaluation of the functional significance of millisecond-range temporal differences.

While the previous discussion pertains mostly to instrumentation, modelling of the signal sources probably plays an increasingly important role in the future. So far, current dipoles with no amplitude priors have been used almost exclusively as the model for each source, but more detailed, biophysically and physiologically inspired generative models could be more informative of the underlying neural processes. These multilevel models could facilitate the much-needed bridging of spatial scales as they could integrate detailed descriptions of single neurons, models of population activity and non-invasive macroscopic measurements.

Inverse modelling algorithms could benefit from full Bayesian approaches that not only integrate information from multiple imaging methods but also allow the estimation of source locations, source time series and even functional networks at a single step to alleviate problems with limited spatial resolution. Should the improvements in instrumentation provide much higher signal-to-noise ratios than available today, the inverse models could also estimate the extent of the active brain areas.

In the future, MEG and EEG may not be the only non-invasive ways of measuring electric signalling of neurons. It has been suggested that, for example, neural currents could generate an MRI contrast by dephasing the spins due to the local neuromagnetic fields. While such direct neuronal current imaging is a very tempting approach as it does not suffer from an ill-posed inverse problem as MEG and EEG do, the effects are so weak (currently perhaps two orders of magnitude below what can be measured) that their detection will remain extremely challenging. In addition, a long acquisition time will probably be needed for detecting these subtle changes, which renders the temporal resolution poor even though one is recording a reflection of the fast neuronal events.

Future brain research may also take advantage of recording devices implantable on the cortex to extract signals for controlling, for example, a prosthetic limb (for a review see [48]). These invasive brain–machine interfaces may develop rapidly and bring useful by-products to basic neuroscience as well. Moreover, recent advantages in spintronics have enabled miniaturized yet highly sensitive magnetic field detectors that operate at body temperature and can record neural activity right on the cortex [49]. Similar sensors can also be integrated on needle-like probes (*d* < 100 µm) that can be inserted into the cortex. The likely benefit of magnetic measurement versus electric recording of local neural activity is increased spatial selectivity, i.e. the ability to attribute the signals to specific neurons, as well as the higher sensitivity to intracellular than extracellular currents of intact neurons.

The contribution of action potentials to these micro- and meso-scale measurements of the future will probably be much larger than it is to MEG and EEG. Therefore, these invasive techniques will provide not only a spatially sharper but also physiologically complementary picture of brain activity with respect to MEG/EEG. An important advantage will be the possibility to look at the timing of single spikes at sub-millisecond resolution.

## 5. Brains in interaction

One can criticize the current brain imaging, with all its great advances, as representing ‘spectator science’ with the assumption that humans (and their brains) are only reactive, so that the ongoing state-changes triggered by the interacting partner, or the physical environment, are not taken into account. Instead, the subject is exposed to a range of crafted stimuli from checkerboard patterns to movies. In reality, however, people are actively participating in the events, not only observing them.

Although social interaction is among the most complex functions that humans (and their brains) perform, it appears surprisingly easy. For example, during conversation, turns are usually taken effortlessly, smoothly and in a temporally accurate manner so that, over different languages and cultures, the gaps between the turns are typically only a few hundred milliseconds, or even less [50]. Such brief intervals cannot reflect just reactions to the end of the previous speaker's utterance; instead the conversation participants have to be synched so that they can unconsciously predict when the other speaker is going to finish their turn at talking.

We have proposed ‘two-person neuroscience’ (2PN) as an approach to study the physiological basis of social interaction [51]. One of the main experimental goals of 2PN could be to differentiate interactive [52] versus reactive states of human social interaction by measuring brain signals from dyads instead of single actors. How big the gain will be from the investments to these rather complicated simultaneous measurements of two persons remains to be seen in the future. Anyhow, 2PN set-ups seem necessary for studies of real social interaction whenever people mutually regulate their dynamic coupling, co-adapting their behaviours. Whether we should take the 2PN approach instead of the easier and more controlled 1PN approach (a spectator view) depends essentially on the timing of the behaviour of interest; phenomena where information is exchanged between the participants at time intervals shorter than 100 ms would need time-resolved brain imaging methods, such as MEG or EEG.

Motivation for two-person ‘hyperscanning’ also derives from the fact that the human brain and mind are shaped from cradle to grave by the interaction with other people. It is even thought that sociality has drastically driven the evolution of the human brain [53]. Importantly, sociality is a group property emerging in relation to other people. From the experimental point of view, it is evident that without measuring two persons at the same time the natural social interaction is difficult or impossible to tackle because the interaction sequences cannot be repeated so that the subjects' brain activity could be measured sequentially.

For 2PN purposes, we have recently built a set-up for simultaneous MEG-to-MEG recordings between two sites. The first measurements were carried out between laboratories 5 km apart in Finland [54], and more recently experiments have been run between our laboratory in Finland and another similarly equipped laboratory in Belgium. The main advantage of MEG over EEG in this kind of 2PN recordings is that the sources of the signals (e.g. of modulation of brain rhythms) can be more accurately identified.

Still, the analysis of 2PN data is extremely challenging. Several approaches are possible. One can compute hyperconnectivity, a measure of functional connectivity between the two brains (instead of estimating connectivity between areas of the same brain). Especially interesting would be to track the time-varying modulations of the connectivity. Intersubject correlations (earlier studied with both fMRI and MEG between subjects who were successively viewing the same movie, e.g. [55–58]) can in the 2PN setting also inform about contextual features typical to that situation only. When data are available from both brains, we can look for correlations between the two sets of brain signals, without explicit reference to the external events.

Combining behavioural and body-state parameters to the analysis (e.g. data from motion-capture or accelerometer sensors, or various measures of the autonomous nervous system function including for example heart rate and its variability) will inform how the brain and body act in synchrony, both within one person and between the two subjects involved in social interaction.

## 6. Timewise insights into brain function

It is now increasingly realized that in addition to the connectome, the structural architecture of the brain, information about neuronal timing is quintessential for understanding the human brain function as well as the behaviour as an emergent property of the brain as a whole. Neurodynamics has even been stated to be the focus of ‘postconnectome science’ [59], meaning that it is not enough to treat the nodes of neural networks solely as places of some activity; instead, information is needed about the local dynamics of the nodes as such, as well as their relative timings.

To draw attention to the importance of timing in brain function, Kopell and co-workers [59] recently introduced the *dynome* as the collection of both experimental and modelling data dealing with the dynamical structure in the brain and its relation to cognition. Similarly, Calhoun *et al*. [60] introduced the *chronnectome*, where functional connectivity may be temporarily changing.

Improved recording accuracy also brings up new questions. The Defense Advanced Research Projects Agency (DARPA) of the USA is, according to media information, considering single-neuron recording from ‘behaving soldiers’ in the field. What would such information tell? How many neurons should we measure to learn something about the percepts or aims of the person? How would we find these cells? How informative would single neurons be as correlates to behaviour?

And what if we had transparent human brains, similar to the mouse brain treated with the CLARITY technique [61]? How much would it help if we were able to measure and follow each action potential and each synaptic activation? Would it be acceptable to integrate the functions of all neurons, interneurons and glial cells in small volumes of brain tissue, such as cortical columns? How should we simplify the models to make them computationally tractable yet physiologically meaningful? Are all synapses and neurons essential, or are they important only occasionally in certain contexts? In other words, would it really be necessary to simulate each single neuron, or should one immediately proceed to a much larger grain level and then rely on the self-organization of the extensive training and plasticity of the units and their connections?

Apparently the problem is not the laborious collection of data, but making sense of them. For that purpose, we definitely should improve the theoretical framework and have models that could be used to make temporo-spatial predictions of new data (even though we are still missing any real ‘brain theory’, perhaps except for the idea that the brain develops and works like a Bayesian inference system) and to make a link from elements to circuits and behaviour.

If we build models with all details—even surviving the abundance of big data [62]—the models will be as difficult to understand as the original biological systems. Thus, we need to simplify down to a level of an analysis unit that is proper for each complexity level. One guideline might be to change from one level to the next whenever (and only when) some emergent properties arise in the system. Emergence itself is a dynamical process, which does not exist in static systems.

An example of a well-known connectome is the structural architecture of the *Caenorhabditis elegans* nervous system, comprising 302 neurons. This structure has been known for a quarter of century and is extensively studied to find out how neural circuitry arises from the interconnected neurons [63]. In such a well-understood system, it might also be possible to find out how much the properties of the neurons can be simplified without losing the emergent properties of the whole circuitry. Ongoing megaprojects in the USA focus on development of new technology that would allow the study, in small mammals, of mesoscopic circuitries that we currently know very little about. Of course, the questions related to the details of the elements and the emergent properties of the networks can be also studied with simulations provided that the temporo-spatial details of the system are fully known.

When pondering about the proper analysis unit, it is good to remember that quite complex behaviour is possible with very simple architecture. For example, ‘Breitenberg vehicles’ [64], simple robots with only uncomplicated control circuits (such as, when a light sensor detects a wall, turn away from the wall, etc.), result in behaviour that for an outside observer seems intelligent. At a little more advanced level, many children, and even adults, are fascinated by movements of robotic vacuum cleaners that they consider intentional.

## 7. Predicting versus creating the future

Predicting the future is not a rewarding task. However, the future is not totally unwritten but rather shaped by the progress until the present moment and by the plans that people have for the future. Single researchers and serendipitous discoveries can quickly change the main direction of science. With the large crowd of scientists some main directions of discoveries can be guessed and even directed by, for example, funding policies and inspiring role-model scientists. The predictability of research directions also increases as more and more research is made in larger units that require considerable investments. In trying to win the necessary funding, many scientists start to behave as opportunists; bees fly where the nectar is, which again increases the power of the funding agencies in directing the science.

The best means to predict the near future is to extrapolate from the past; for example, by predicting tomorrow's weather from that of today as the weather conditions of two successive days are highly correlated. But predictions of future innovations are stated to often overestimate short-term developments and to underestimate long-term changes.

It is expected that in the years to come, old ideas and research questions will reappear and be tested with more rigorous and accurate methods. For example, the relationship between behaviour and brain function can be assessed more accurately with the improved instrumentation and better integration of multilevel temporo-spatial information and modelling covering multiple temporal and spatial scales. Big data are already now changing the way we do research, and data-driven machine learning approaches are likely to continue to advance our understanding of human brain function.

We would like to see the development of a solid theoretical framework for human brain function and more understanding of brain dynamics in naturalistic situations, as well as measurements of multiple persons at the same time. When we move from the laboratory environment to naturalistic conditions, the picture of brain function may change as dramatically as happened when it first became possible to make neurophysiological recordings from awake animals and humans in contrast to the previous experiments limited to anaesthetized animals.

The focus of current brain imaging has been much on healthy (student) populations, but should be equally broadened to all age groups. Time-resolved brain imaging may be especially suitable for the study of brain development from neonates to adolescents, when the transmission delays change drastically as a function of increased myelination and changes in neuronal transmission. Moreover, it would be beneficial to study more extensively subjects with special abilities, and not only patients with damaged brains.

Finally, brain function does not explain everything about human behaviour and mind. One has to consider the body, environment and culture as well.
